# Study on the Elastic–Plastic Correlation of Low-Cycle Fatigue for Variable Asymmetric Loadings

**DOI:** 10.3390/ma13112451

**Published:** 2020-05-28

**Authors:** Junhong Zhang, Weidong Li, Huwei Dai, Nuohao Liu, Jiewei Lin

**Affiliations:** 1 State Key Laboratory of Engines, Tianjin University, Tianjin 300072, China; zhangjh@tju.edu.cn (J.Z.); liwd@tju.edu.cn (W.L.); dhwmail@tju.edu.cn (H.D.); knowhow@tju.edu.cn (N.L.); 2 Renai College, Tianjin University, Tianjin 301636, China

**Keywords:** low cycle fatigue, life prediction, strain ratio, mean stress, asymmetric loading

## Abstract

The mean stress effect in fatigue life varies by material and loading conditions. Therefore, a classical low cycle fatigue (LCF) model based on mean stress correction shows limits in asymmetric loading cases in both accuracy and applicability. In this paper, the effect of strain ratio (R) on LCF life is analyzed and a strain ratio-based model is presented for asymmetric loading cases. Two correction factors are introduced to express correlations between strain ratio and fatigue strength coefficient and between strain ratio and fatigue ductility coefficient. Verifications are conducted through four materials under different strain ratios: high-pressure tubing steel (HPTS), 2124-T851 aluminum alloy, epoxy resin and AZ61A magnesium alloy. Compared with current widely used LCF models, the proposed model shows a better life prediction accuracy and higher potential in implementation in symmetric and asymmetric loading cases for different materials. It is also found that the strain ratio-based correction is able to consider the damage of ratcheting strain that the mean stress-based models cannot.

## 1. Introduction

Under high-amplitude cyclic loads, the fatigue process of materials is dominated by plastic strain that results in a short fatigue life in loading cycles, so-called low-cycle fatigue (LCF). It is well known from the monotonic tensile curve that the stress grows slowly while the strain increases rapidly when the stress reaches the yield limit. Therefore, either the experimental or the analytical study on the LCF uses the strain control method. When the R ≠ −1, the non-zero mean stress caused by the asymmetric loading cycles will affect the fatigue process considerably while the tensile mean stresses are detrimental to fatigue life while the compressive mean stresses are beneficial to fatigue life [1,2]. Engineering structures suffered from fatigue failure usually bears asymmetric cyclic loading rather than a symmetric one. Therefore, the prediction of fatigue life requires an understanding of material behavior under non-zero mean stress/strain conditions. This is why this sort of mean stress effect on the fatigue behavior of material has been widely studied in the past few decades. As outputs, a large number of mean stress models have been proposed to correct the fatigue life curve.

Early studies considered that mean strain is ineffective on the fatigue resistance unless it can result in non-zero mean stress during cyclic loading. The authors in [3] believe that the mean strain has little influence on fatigue, but the mean stress has a significant effect. Under this consideration, the Goodman [4] mean stress correction is widely used in engineering applications. Soderberg [5] used yield strength to replace the tensile limit in the Goodman mean stress correction to tune the prediction conservative. Morrow [6] considered the mean stress as the main contribution on the elastic region of fatigue and used the fatigue strength coefficient $\sigma_{f}^{\prime }$ to replace the tensile limit in the Goodman mean stress correction to propose the model. The Morrow model shows good predictions when the fatigue strength coefficient is close to fracture strength, otherwise the prediction of Morrow model is not convincible. The Smith–Watson–Topper (SWT) model [7] based on the energy concept defines the product of strain amplitude and maximum stress as damage parameter considering that the maximum stress dominates the influence of mean stress on fatigue life. Lv [8] introduced the Walker exponent into the SWT model to describe the sensitivity of mean stress. Zhang [9] took the mean stress relaxation into account in the SWT model based on Landgraf model to improve the fatigue life prediction. Wang [10] proposed an alternative model based on an equivalent strain amplitude and showed excellent correlations in fatigue life data under different strain ratios of rubber. The authors in [11] show that brittle materials are more sensitive to mean stress than ductile materials. The mean stress sensitivity is affected by cyclic softening, cyclic hardening and mean stress relaxation. So a concept of mean stress sensitivity is incorporated to modify the total strain energy density by introducing two mean stress correction factors. It provides better predictions for 8 materials with lower mean error than SWT, Ince–Glinka [12] and GDP (Generalized energy-based fatigue-creep Damage Parameter) models [13].

It can be seen that most works on LCF models were done on the mean stress correction. However, the mean stress affected by cyclic softening or hardening changes with loading cycles and will lead to errors in life prediction [14,15]. In high cycle fatigue (HCF), the mean stress can be simply calculated by the stress ratio. However, the mean stress and strain amplitude cannot be obtained at the same time, which leads to extra effort in life prediction in engineering applications. In addition, how the strain ratio could affect the mean stress and life prediction depends on loading conditions, i.e., the strain ratio and the strain amplitude. It would be more convenient for LCF life prediction to use a directly controlled factor, such as the strain ratio, rather than an intermediate parameter, such as the mean stress. Wang [11] proposed a strain ratio-based LCF model, of which the model parameters need at least three groups of fatigue data at different strain ratios.

Mean stress-based LCF models usually over-consider the effect of mean stress on fatigue life especially for asymmetric loading case with small amplitude strain. Besides, the mean stress is hard to obtain in the strain-controlled LCF experiments, which limits the applicability of such models. In this paper, the correlation between strain and stress in LCF is analyzed and a strain ratio-based modification on LCF model is proposed for asymmetric loading condition. Linear relationships behind the fatigue strength coefficient, the fatigue ductile coefficient and the strain ratio are developed and employed in the model modification. The proposed LCF model is then verified and compared with other commonly used LCF models. 

## 2. Elastic–plastic Correlation of LCF

**Figure 1:**
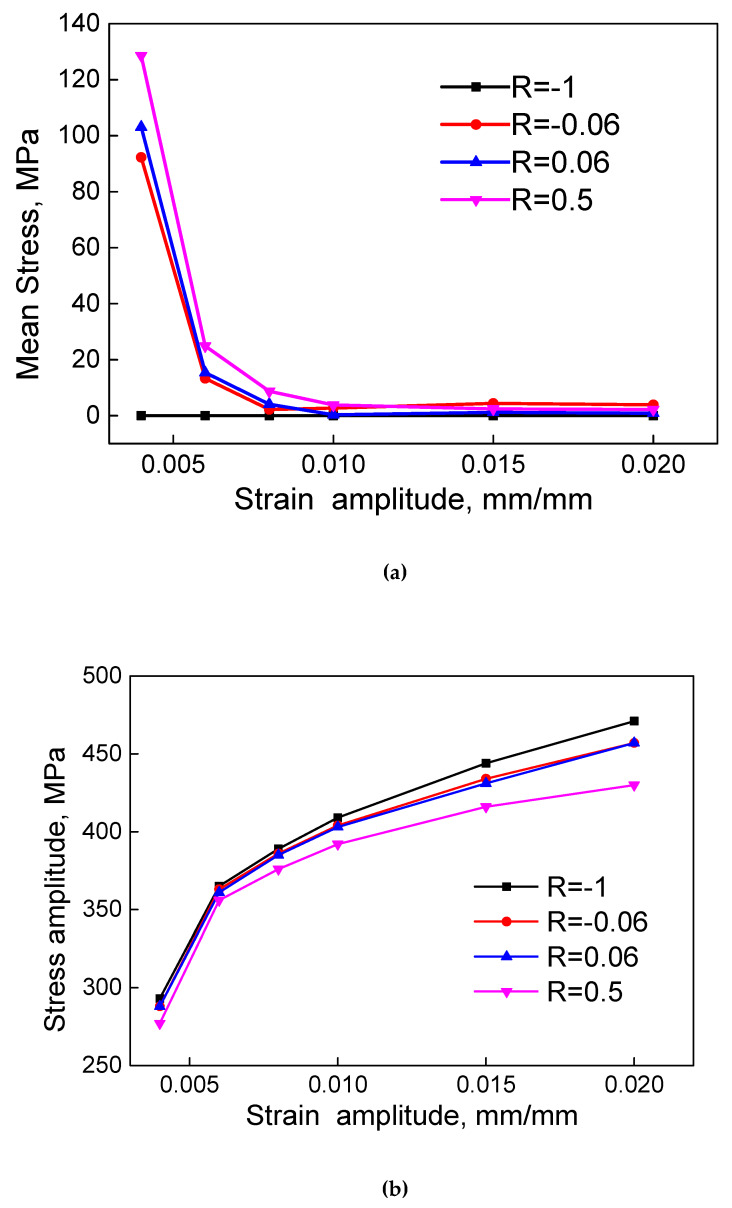
Comparisons on (**a**) mean stress and (**b**) stress amplitude of 2124-T851 aluminum alloy under different strain ratios, *R*.

To recognize the relationship between stress and strain under different strain ratios, experimental data of 2124-T851 aluminum alloy from [16] are presented in Figure 1. The experiment was carried out by strain-controlled method at a constant total strain rate of 0.004 s^−1^ at room temperature. Since the mean stress and the stress amplitude are the two factors usually considered as the control parameters in mean stress-based fatigue models, two diagrams are reproduced as the mean stress versus the strain amplitude (Figure 1a) and the stress amplitude versus the strain amplitude (Figure 1b) under four levels of strain ratios.

For *R* = 0.5, the mean stress decreases with increasing strain amplitude at quite a significant decreasing rate in the low strain amplitude range from 0.005 to 0.01. As the strain amplitude further increases, the decreasing rate of mean stress reduces and the mean stress reaches a stable level above zero when the strain amplitude is greater than 0.01. The same patterns can be found in cases *R* = 0.06 and *R* = −0.06, and the overall mean stress decreases with increasing strain ratio. For *R* = −1, in particular, the mean stress keeps as zero and does not vary with the strain amplitude. The mean stress relaxation rate is the main cause of the variation of mean stress. When the plastic strain raises, the mean stress relaxation rate grows and leads to a sharp drop in mean stress. After that, the relaxation rate of mean stress remains at a relatively high level and the mean stress keeps decreasing as the strain amplitude increases. As we can see, in the small strain range, the mean stress varies a lot for different strain ratios. This effect of strain ratio on the mean stress is not significant for the large strain range.

The stress amplitude increases with increasing strain amplitude, but it decreases with increasing strain ratio from −1 to 0.5. So, under small strain amplitudes, the effect of strain ratio on the fatigue life of the material is reflected in the mean stress and the elastic strain control of the fatigue process. For large strain amplitudes, the plastic strain takes their place and embodies the impact of strain ratio.

**Figure 2:**
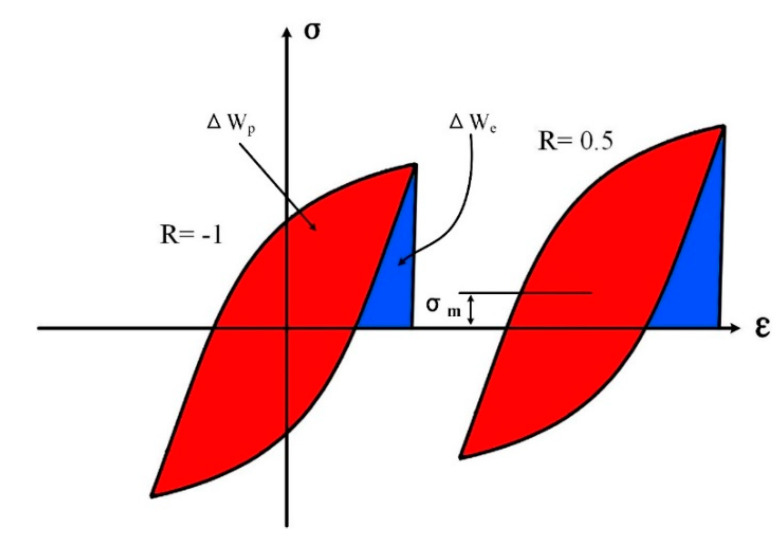
Schematic of hysteretic loops under same strain amplitude and different strain ratios of 0 and 0.5.

Figure 2 shows a comparison of hysteresis loops at different strain ratios. The total strain energy per-cycle can be divided into plastic strain energy Δ*W*_p_ and elastic tensile strain energy Δ*W*_e_. For a fully reversed loading case (*R* = −1), the hysteresis loop is symmetrical according to the coordinate origin, and the mean stress is zero. In the strain ratio *R* = 0.5 case, there is a non-zero mean stress *σ*_m_ during the cyclic loading process. As the mean stress varies, the elastic strain energy Δ*W*_e_ changes by a great deal. So, the elastic strain energy increases with increasing strain ratio at a specific strain amplitude. It is also known that under a specific strain amplitude the stress amplitude decreases with increasing strain ratio. It means the plastic strain energy Δ*W*_p_ decreases as the strain ratio raises and this phenomenon becomes more obvious as the strain amplitude increases. As a result, the mean stress and the cyclic strain softening affect the fatigue life of the material by means of changing the total strain energy, which explains the influence mechanism of strain ratio on fatigue life. 

For small strain amplitudes, there is a strong effect of strain ratio on the mean stress and weak effect on the cyclic strain-softening. So the total strain energy depends on mean stress. For large strain amplitudes, the mean stress is relatively insensitive to the strain ratio, but the cyclic softening effect dominates. That is to say that the strain ratio indeed affects the fatigue process, and the effect of strain ratio embodies different indices according to the strain amplitude.

## 3. LCF Models for Asymmetric Cyclic Loading

### 3.1. Model Description

The Manson–Coffin model, given as Equation (1), is suitable for life predictions under fully reversed loading cases.

(1) $$\varepsilon_{a}=\frac{{\sigma^{\prime }}_{f}}{E}{\left(2N_{f}\right)}^{b}+{\varepsilon^{\prime }}_{f}{\left(2N_{f}\right)}^{c}$$

where $\sigma_{f}^{\prime }$ is the fatigue strength coefficient, *b* the fatigue strength exponent, $\varepsilon_{f}^{\prime }$ the fatigue ductility coefficient, and *c* the fatigue ductility exponent.

In the asymmetric loading condition, the effect of strain ratio is generally considered through equivalent stress, like Goodman model, which is expressed as

(2) $$\frac{\sigma_{a}}{\sigma_{eq}}+\frac{\sigma_{m}}{\sigma_{u}}=1$$

where $\sigma_{a}$ is the stress amplitude, $\sigma_{m}$ the mean stress, $\sigma_{u}$ the tensile limit of material, and $\sigma_{eq}$ the equivalent stress amplitude. Equation (2) can be also expressed as

(3) $$\sigma_{eq}=\frac{\sigma_{a}}{1-\sigma_{m}/\sigma_{u}}=\sigma_{f}{}^{\prime }{\left(2N_{f}\right)}^{b}$$

The Manson–Coffin model is then modified into

(4) $$\varepsilon_{a}=\sigma_{a}/E+\varepsilon_{p}=\frac{{\sigma^{\prime }}_{f}-\left(\sigma_{m}\cdot {\sigma^{\prime }}_{f}\right)/\sigma_{u}}{\cdot E}{\left(2N_{f}\right)}^{b}+{\varepsilon^{\prime }}_{f}{\left(2N_{f}\right)}^{c}$$

Replacing the fatigue strength coefficient ${\sigma^{\prime }}_{f}$ by the tensile limit $\sigma_{u}$ [6], the equivalent stress and the fatigue model will be

(5) $$\sigma_{eq}=\frac{\sigma_{a}}{1-\sigma_{m}/{\sigma^{\prime }}_{f}}$$

(6) $$\varepsilon_{a}=\sigma_{a}/E+\varepsilon_{p}=\frac{{\sigma^{\prime }}_{f}-\sigma_{m}}{\cdot E}{\left(2N_{f}\right)}^{b}+{\varepsilon^{\prime }}_{f}{\left(2N_{f}\right)}^{c}$$

Kwofie [17] considered that, at a specific stress amplitude, a decrease in fatigue life due to mean stress must be led by a corresponding decrease in the fatigue strength coefficient $\sigma_{f}^{\prime }$. The influence is related to the rate of mean stress to tensile limit. The fatigue life decreases with stress in a non-linear manner, and the fatigue crack growth increases with the cycle number, which may be described by an exponential function.

(7) $$\sigma_{eq}=\sigma_{a}e^{\alpha \frac{\sigma_{m}}{\sigma_{u}}}$$

where *α* is a material constant. Walker [18] also proposed an equivalent stress model based on the mean stress as

(8) $$\sigma_{eq}=\sigma_{\max}^{1-\gamma }\sigma_{a}^{\gamma }={\left(\sigma_{a}+\sigma_{m}\right)}^{1-\gamma }\sigma_{a}^{\gamma }$$

where *γ* is a material constant.

The walker model turns into the SWT model while *γ* = 0.5 as Equation (9). The model used in LCF life prediction can be expressed as Equation (10).

(9) $$\sigma_{eq}=\sqrt{\sigma_{\max}\sigma_{a}}$$

(10) $$\sigma_{\max}\varepsilon_{a}=\frac{{\left({\sigma^{\prime }}_{f}\right)}^{2}}{E}{\left(2N_{f}\right)}^{2b}+{\sigma^{\prime }}_{f}{\varepsilon^{\prime }}_{f}{\left(2N_{f}\right)}^{b+c}$$

As we can see, the equivalent stress is the common corrector used in the fatigue model modifications. For the Goodman model and the Morrow model, the equivalent stress only depends on the mean stress. However, the Kwofie model and the Walker model use the mean stress *σ*_m_ and the stress amplitude *σ*_a_ to calculate the equivalent stress together.

**Figure 3:**
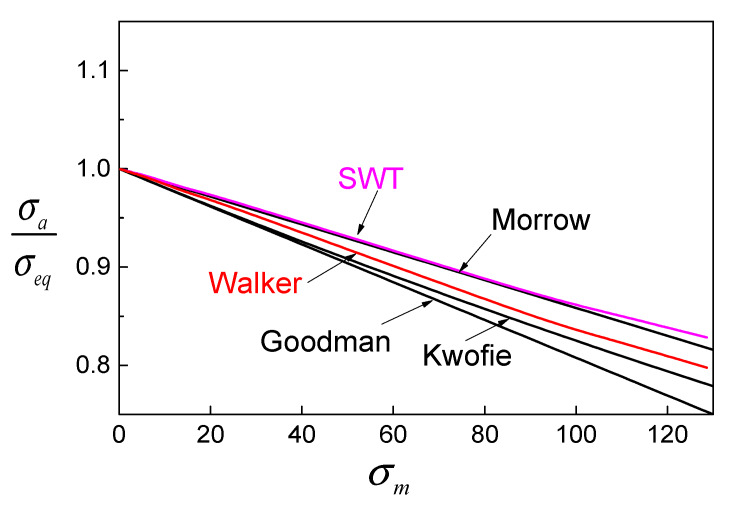
Comparison of different modifications of fatigue models.

A dimensionless ratio of *σ*_a_*/σ*_eq_ is employed to compare these modifications, which can represent the relationship between fatigue life and mean stress indirectly. The reason is that, at a given stress amplitude *σ*_a_, a higher equivalent stress *σ*_eq_ results in a lower fatigue life. So, Figure 3 reflects how the modifications on fatigue life change according to the mean stress of different fatigue models. An obvious trend can be found in that *σ*_a_*/σ*_eq_ decreases with the increase of mean stress *σ*_m_. When the mean stress is small, *σ*_a_*/σ*_eq_ of all models are close to each other, and the gap between models grows with increasing mean stress. The Goodman model has the lowest *σ*_a_*/σ*_eq_ and will lead to the lowest fatigue life. The predicted lives of the Walker and Kwofie models depend on parameters *α* and *γ*. Using the Walker model and the Kwofie model can improve the life prediction, but this depends on the determination of *α* and *γ*, which could be uncertain in practice.

Lv [8] considered that the effect of mean stress on fatigue life varies from materials can be represented by mean stress sensitivity. For the SWT model, the mean stress sensitivity is a constant of 0.5 and deviations will lead to errors in the life prediction. The material constant of Walker model, *γ*, can express the mean stress sensitivity as well. So, the SWT model can be modified into Equation (11).

(11) $$2\gamma \sigma_{\max}\varepsilon_{a}=\frac{{\left({\sigma^{\prime }}_{f}\right)}^{2}}{E}{\left(2N_{f}\right)}^{2b}+{\sigma^{\prime }}_{f}{\varepsilon^{\prime }}_{f}{\left(2N_{f}\right)}^{b+c}$$

Other than the mean stress, Wang [10] used an equivalent strain amplitude to incorporate the effect of strain ratio into life prediction. The equivalent strain amplitude is defined as

(12) $$\varepsilon_{eq}={\left(1+\beta \frac{1+R}{1-R}\right)}^{n}\cdot \varepsilon_{a}=A\cdot N_{f}{}^{b}$$

where *β* and *n* are material constants.

### 3.2. Model Comparison

**Figure 4:**
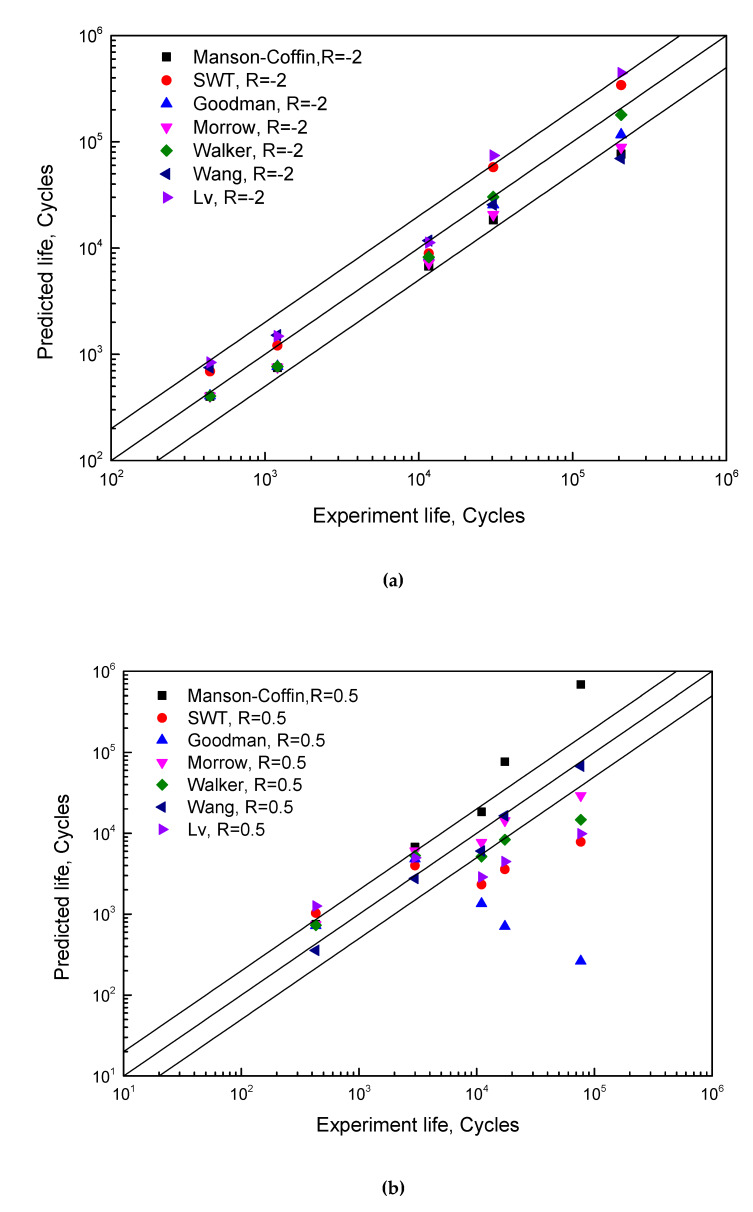
Comparisons between fatigue models under strains of (**a**) *R* = −2 and (**b**) *R* = 0.5.

Fatigue data of high-pressure tubing steel (HPTS) [19] under strain ratios of −2, and 0.5 are used to compare the abovementioned fatigue models, as shown in Figure 4. 

The effect of mean stress on fatigue life depends on the direction. For a tension–compression case (*R* < 0), the compressive stress has a beneficial effect by extending the fatigue life, while the tensile stress takes the main response for the fatigue damage accumulation. Under this consideration, a lower strain ratio means a larger compressive stress, which leads to a longer fatigue life, and vice versa. The Manson–Coffin model does not consider the mean stress effect, so the life prediction will be higher than the experimental data for *R* = 0.5 due to the existence of tensile mean stress. Aside from the Manson–Coffin model, other models show a common feature that the predictions are smaller than the experiment results in lower strain ranges. This is because the mean stress corrections in these models are over-considered when the mean stress raises rapidly with decreasing strain amplitude. In particular, for mean stress-based models, the deviation of prediction is strongly affected by the correction intensity of mean stress as shown in Figure 3. It is why the Goodman model has the lowest predictions and the Morrow model gives relatively high results.

The Lv model is a transformation of the SWT model, introducing a material constant γ from the Walker model to consider the sensitivity of mean stress. When γ > 0.5, the prediction life of Lv model is larger than the SWT model. Therefore, the prediction accuracy of Lv model is worse than the SWT model for R = −2 and vice versa for R = 0.5. This means that fitting different γ values according to different strain ratios are essential for Lv model to provide accurate predictions. However, the material constant, γ, is not defined as a variable in the Walker model, which means γ has a determined value for a certain material no matter how the loading condition changes, i.e., for different strain ratios. As a result, the Lv model, which inherits γ from the Walker model, cannot fit for all strain ratios. In other words, an Lv model with higher γ value is suitable for the low strain ratio case, while that with smaller γ value can predict the high strain ratio case better. Using the equivalence strain amplitude, Wang’s model gives the best predictions in asymmetric loading cases. 

The mean stress-based models mainly focus on the elastic strain, and the predictions usually excessively consider the effect of mean stress. The Walker model and the Lv model weaken the above over-consideration by introducing the sensitivity coefficient of mean stress, but they sacrifice in terms of feasibility. In addition, the mean stress relaxation, cyclic softening and cyclic hardening will affect the mean stress and influence the prediction accuracy. Thanks to the strain ratio-based modification, the Wang model can avoid the interferences from stress relaxation and cyclic softening/hardening and offers acceptable predictions under asymmetric loading cases. It can be concluded that the strain ratio-based fatigue model is more appropriate for the strain-controlled LCF case.

## 4. Model Modification

### 4.1. Modification

To predict fatigue life, the Manson–Coffin model needs to obtain four parameters: the fatigue strength coefficient $\sigma_{f}^{\prime }$, the fatigue strength exponent *b*, the fatigue ductility coefficient $\varepsilon_{f}^{\prime }$, and the fatigue ductility exponent *c*. Parameters *b* and *c* represent the slopes of the elastic and plastic shares in the fatigue process, $\sigma_{f}^{\prime }$ and $\varepsilon_{f}^{\prime }$ represent the intercepts of the elastic and plastic shares on the coordinate axis. Ong [20] analyzed the fatigue behaviors of 49 metals using the Manson–Coffin model and proved that *b* and *c* are related to the tensile limit, the true fracture strength and the true fracture ductility from the monotonic tensile test. As a result, these two parameters are independent from strain ratio. The Manson–Coffin model can be transformed into

(13) $$\varepsilon_{a}=2^{b}\cdot \frac{{\sigma^{\prime }}_{f}}{E}N_{f}{}^{b}+2^{c}{\varepsilon^{\prime }}_{f}N_{f}{}^{c}$$

where $2^{b}\cdot \sigma_{f}^{\prime }/E$ and $2^{c}\cdot \varepsilon_{f}^{\prime }$ are the Elastic strain amplitude and plastic strain amplitude of the hysteresis loop for one cycle failure, respectively. The total strain energy required for failure is a certainty [21]. The change of strain ratio leads to a change in the proportion of elastic strain energy and plastic strain energy for one cycle failure. Accordingly, the strain ratio will affect the values of $\sigma_{f}^{\prime }$ and $\varepsilon_{f}^{\prime }$. However, most current fatigue models only focus on $\sigma_{f}^{\prime }$, and the lack of consideration for $\varepsilon_{f}^{\prime }$ may have adverse effects on the life prediction, especially for high-amplitude strain.

According to Equation (6), the modifications are mainly made on the fatigue strength coefficient using the mean stress as

(14) $$\varepsilon_{a}=\sigma_{a}/E+\varepsilon_{p}=\frac{{\sigma^{\prime }}_{f}+f\left(\sigma_{m}\right)}{\cdot E}{\left(2N_{f}\right)}^{b}+{\varepsilon^{\prime }}_{f}{\left(2N_{f}\right)}^{c}$$

where *f*(*σ*_m_) is the function of mean stress.

For the strain-controlled LCF, the mean stress of material is caused by the asymmetric strain, so Equation (14) can be transformed into

(15) $$\varepsilon_{a}=\sigma_{a}/E+\varepsilon_{p}=\frac{{\sigma^{\prime }}_{f}+f\left(R\right)}{\cdot E}{\left(2N_{f}\right)}^{b}+{\varepsilon^{\prime }}_{f}{\left(2N_{f}\right)}^{c}$$

The plastic strain is affected by the strain ratio, in large strain amplitude range in particular, so the fatigue ductility coefficient needs to be corrected by the strain ratio as below.

(16) $$\varepsilon_{a}=\frac{\sigma_{f}{}^{\prime }+f\left(R\right)}{E}\cdot {\left(2N_{f}\right)}^{b}+\left(\varepsilon_{f}{}^{\prime }+g(R)\right)\cdot {\left(2N_{f}\right)}^{c}$$

where *f*(*R*) is the corrected fatigue strength coefficient, and *g*(*R*) is the corrected fatigue ductility coefficient. 

**Figure 5:**
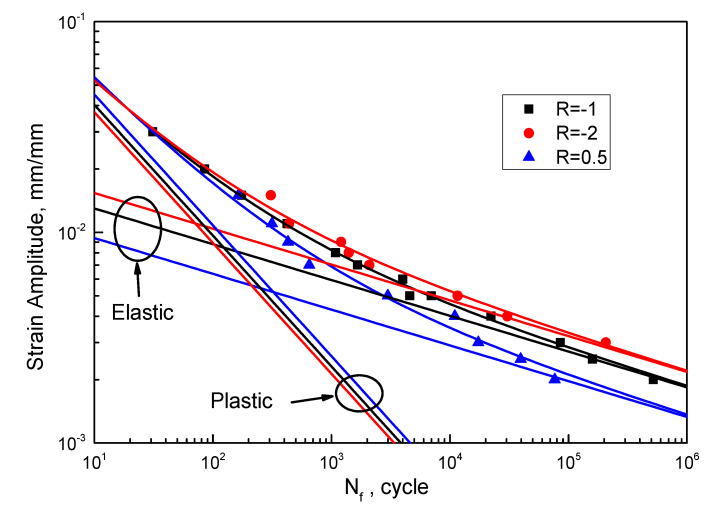
LCF lives of HPTS under different strain ratios.

To study the effect of strain ratio on *f*(*R*) and *g*(*R*), a correlation analysis is performed. Equations (17)–(19) are fatigue life expressions fitted by the HPTS data, in which Equation (17) is fitted based on the Manson–Coffin model, and Equations (18) and (19) are fitted using the proposed model (Equation (16)). The fitting curve is shown in Figure 5.

(17) $$\varepsilon_{a}=0.01919\cdot {\left(2N_{f}\right)}^{-0.1698}+0.1682\cdot {\left(2N_{f}\right)}^{-0.6207}(R=-1)\;r^{2}=0.9994$$

(18) $$\varepsilon_{a}=0.02273\cdot {\left(2N_{f}\right)}^{-0.1698}+0.1552\cdot {\left(2N_{f}\right)}^{-0.6207}(R=-2)\;r^{2}=0.9960$$

(19) $$\varepsilon_{a}=0.01389\cdot {\left(2N_{f}\right)}^{-0.1698}+0.1872\cdot {\left(2N_{f}\right)}^{-0.6207}(R=0.5)\;r^{2}=0.9928$$

**Figure 6:**
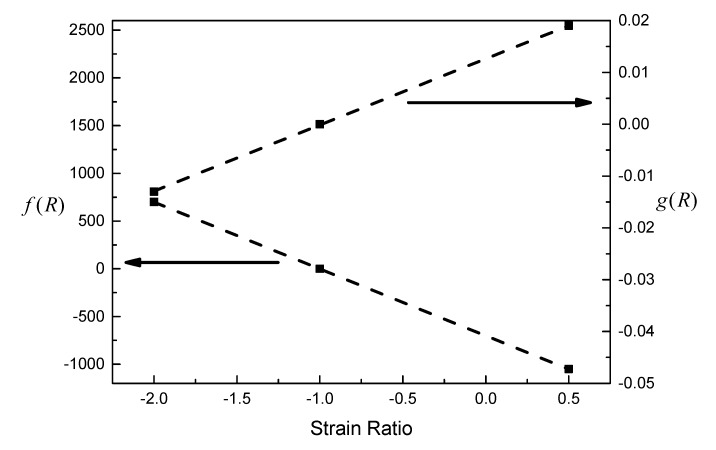
Relationships between *f*(*R*) and *g*(*R*) and strain ratio.

It can be seen from Figure 5 that the prediction curve fits well with the experimental data, and the slopes of the elastic and plastic lines do not change with strain ratio. As shown in Figure 6, the corrected fatigue strength coefficient, *f*(*R*), and the corrected fatigue ductility coefficient, *g*(*R*), are both linearly correlated with the strain ratio.

(20) $$f\left(R\right)=k_{\sigma }\cdot \left(R+1\right)$$

(21) $$g(R)=k_{\varepsilon }\cdot \left(R+1\right)$$

where $k_{\sigma }$ and $k_{\varepsilon }$ are material constants, which are adjustable based on the sensitivity to the strain ratio. The fatigue life of the material will change rapidly if the absolute values of $k_{\sigma }$ and $k_{\varepsilon }$ vary.

Then, Equation (16) can be transformed as follows,

(22) $$\varepsilon_{a}=\frac{\sigma_{f}{}^{\prime }+k_{\sigma }\cdot \left(R+1\right)}{E}\cdot {\left(2N_{f}\right)}^{b}+\left[\varepsilon_{f}{}^{\prime }+k_{\varepsilon }\cdot \left(R+1\right)\right]\cdot {\left(2N_{f}\right)}^{c}$$

For the fully reversed loading case (*R* = −1), the correction values $k_{\sigma }$ and $k_{\varepsilon }$ are 0, the model degenerates to the Manson–Coffin model. 

### 4.2. Verification

**Figure 7:**
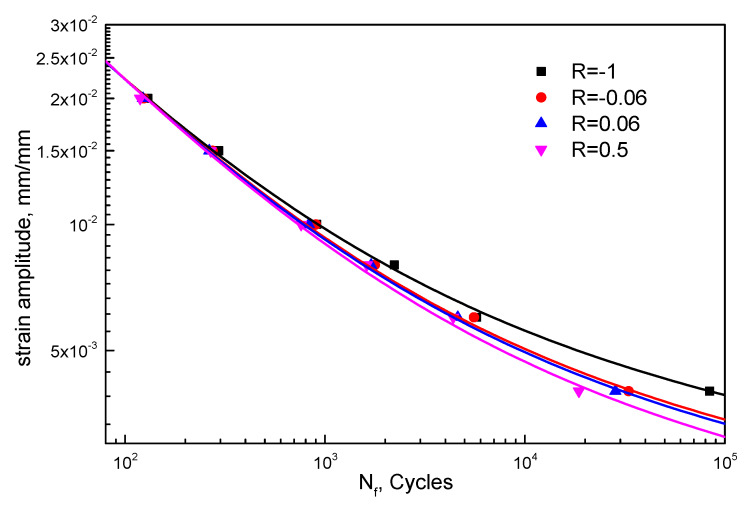
LCF lives of 2124-T851 under different strain ratios.

**Figure 8:**
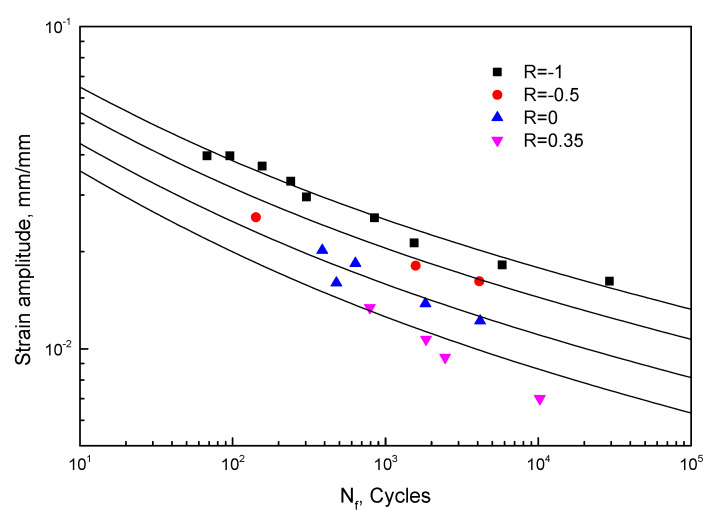
Low-cycle fatigue (LCF) lives of epoxy resin under different strain ratios.

**Figure 9:**
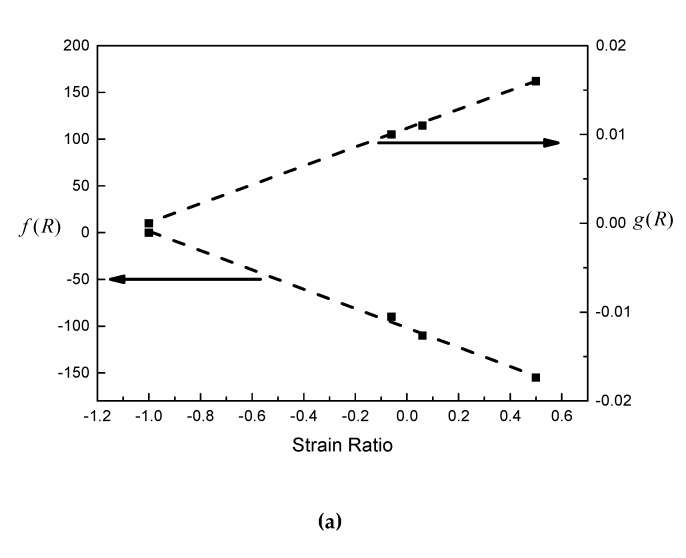

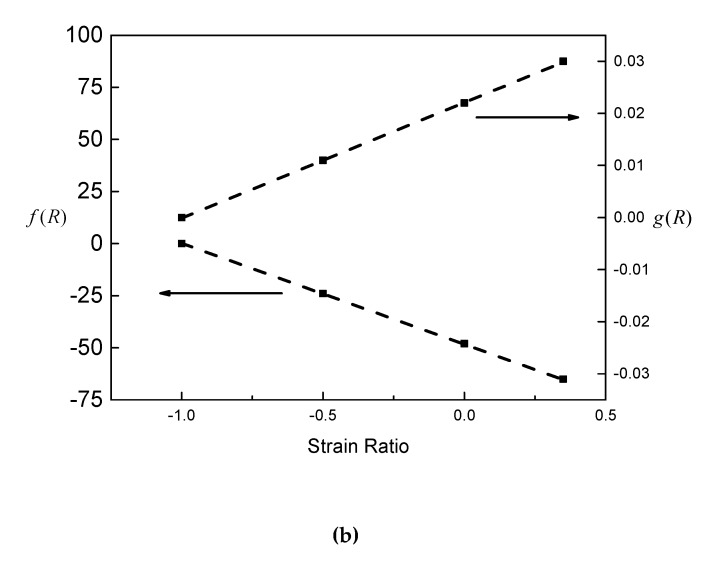
Relationship between correction value and strain ratio of (**a**) 2124-T851 aluminum alloy and (**b**) epoxy resin.

Prior to the prediction accuracy, the basis of the proposed model modification, the linear functions between *f*(*R*) and *g*(*R*) and the strain ratio, are examined. Fatigue data of different materials under various strain ratios are employed, including T851 from [22] and epoxy resin from [23]. The fatigue strength coefficient $\sigma_{f}^{\prime }$, the fatigue strength exponent *b*, the fatigue ductility coefficient $\varepsilon_{f}^{\prime }$, and the fatigue ductility exponent *c* are obtained using the *R* = −1 data. The fitting curves of 2124-T851 and epoxy resin are shown in Figure 7 and Figure 8, respectively. Equations (23)–(26) are fatigue life expressions for 2124-T851, and Equations (27)–(30) are for epoxy resin. Then, *f*(*R*) and *g*(*R*) are calculated by data under other strain ratios. As shown in Figure 9, the linear relationships between *f*(*R*) and *g*(*R*) and the strain ratio are quite clear even for different materials.

(23) $$\varepsilon_{a}=0.00828\cdot {\left(2N_{f}\right)}^{-0.075}+0.188\cdot {\left(2N_{f}\right)}^{-0.53}(R=-1)\;r^{2}=0.9995$$

(24) $$\varepsilon_{a}=0.00706\cdot {\left(2N_{f}\right)}^{-0.075}+0.198\cdot {\left(2N_{f}\right)}^{-0.53}(R=-0.06)\;r^{2}=0.9993$$

(25) $$\varepsilon_{a}=0.00687\cdot {\left(2N_{f}\right)}^{-0.075}+0.199\cdot {\left(2N_{f}\right)}^{-0.53}(R=0.06)\;r^{2}=0.9996$$

(26) $$\varepsilon_{a}=0.0063\cdot {\left(2N_{f}\right)}^{-0.075}+0.204\cdot {\left(2N_{f}\right)}^{-0.53}(R=0.5)\;r^{2}=0.9987$$

(27) $$\varepsilon_{a}=0.04207\cdot {\left(2N_{f}\right)}^{-0.1052}+0.082\cdot {\left(2N_{f}\right)}^{-0.41}(R=-1)\;r^{2}=0.9879$$

(28) $$\varepsilon_{a}=0.03379\cdot {\left(2N_{f}\right)}^{-0.1052}+0.071\cdot {\left(2N_{f}\right)}^{-0.41}(R=-0.5)\;r^{2}=1$$

(29) $$\varepsilon_{a}=0.02552\cdot {\left(2N_{f}\right)}^{-0.1052}+0.06\cdot {\left(2N_{f}\right)}^{-0.41}(R=0)\;r^{2}=0.9041$$

(30) $$\varepsilon_{a}=0.01966\cdot {\left(2N_{f}\right)}^{-0.1052}+0.052\cdot {\left(2N_{f}\right)}^{-0.41}(R=0.35)\;r^{2}=0.9932$$

It can be considered that $k_{\sigma }$ and $k_{\varepsilon }$ are constants for a certain material. By obtaining $k_{\sigma }$ and $k_{\varepsilon }$ by two groups of tests with different strain ratios, the proposed model can be used in any other strain ratios without obtaining the mean stress, which is a great advantage in the application of life prediction for asymmetric loading cases. 

**Figure 10:**
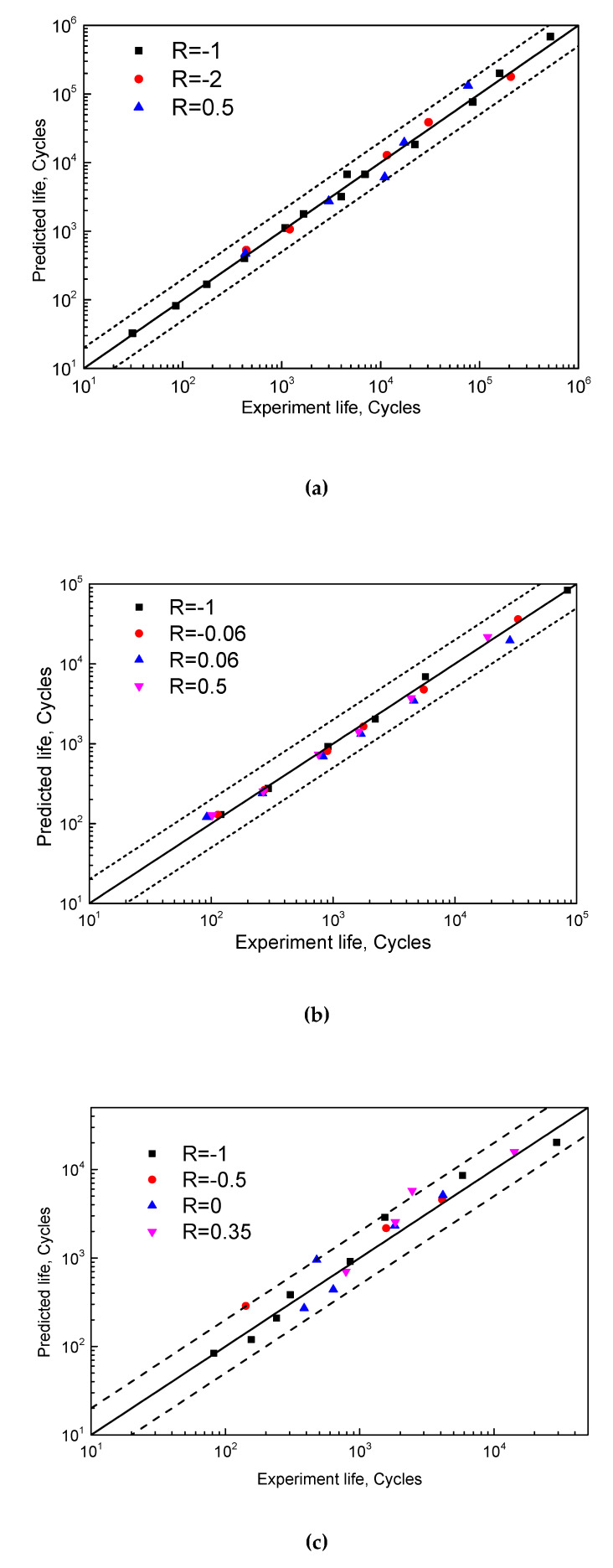
Prediction performance of proposed model for (**a**) HPTS, (**b**) T851 and (**c**) epoxy resin.

The proposed model is employed to predict the fatigue life of the HPTS, T851 and epoxy resin and results are shown in Figure 10. The relative errors of the predicted lives fall within the double scatter band, which is much better than most of the models mentioned in Figure 2.

## 5. Discussions

A dimensionless constant *z* is defined as

(31) $$z=\frac{N_{p}-N_{f}}{N_{f}}=\frac{N_{p}}{N_{f}}-1$$

where *N_p_* is the predicted life and *N_f_* is the experimental life. Since (*N_p_*–*N_f_*) is a measure of the discrepancy of predicted life and experiment life, the r.m.s. of *z*, *S_z_*, can represent the discrepancy level of predictions.

(32) $$S_{z}=\sqrt{\frac{\sum_{i=1}^{k}z_{i}{}^{2}}{k}}$$

where *k* is the number of scattered points. The discrepancy level *S_z_* of the predictions of 8 models for four sorts of material are calculated and listed in Table 1.

Apparently, the life prediction of the Manson–Coffin model shows the largest relative error since it does not consider the effect of strain ratio, while other models show better performance no matter what correction criteria they follow. The effect of strain ratio on the mean stress relaxation rate of T851 aluminum alloy is smaller compared with that of other materials, for small loading cases in particular. This is the reason why the prediction discrepancy of T851 aluminum alloy is smaller than HPTS and epoxy resin for all models. The Morrow model and Walker model are more accurate in HPTS due to the smaller correction intensity in small strain cases as shown in Figure 4. The SWT model defines a damage parameter of $\varepsilon_{a}\sigma_{max}$ to correct the effect of mean stress on fatigue life, and it overestimates the effect in large mean stress cases [24]. This is why considerable discrepancies in predictions are found for HPTS in large mean stress conditions. The Lv model shows a better prediction than the SWT model because it considers the sensitivity of mean stress. All models show big gaps in the epoxy resin, particularly for the Goodman model and Morrow model. One reason is that epoxy resin has anisotropic behavior in tension and compression, which affects the mean stress in cyclic loading process; therefore, the mean stress cannot fully reflect the effect of strain ratio on damage. The other reason is that considerable ratcheting strain could be accumulated in fatigue tests due to the viscoelastic behavior of polymer materials, and a certain amount of irreversible strain energy will be converted into heat dissipation or cause damage that cannot be described by the mean stress. In other words, models based on mean stress are not capable of accurately predicting the fatigue life of materials with large ratcheting strain. The ratcheting strain is more obvious for non-metallic materials than metals, but the temperature will intensify the ratcheting strain of metals. That is to say, the mean stress-based model may not be available for non-metallic materials and high-temperature metals. The Wang model performs well in non-metallic materials due to the correction of strain ratio. In other words, strain ratio-based correction is more suitable for asymmetric loading cases. This is why the proposed model adds the correction term to the fatigue strength coefficient and fatigue ductility coefficient, which can express the influence of the strain ratio and viscoelastic behavior of the material. 

## 6. Conclusions

In this paper, the correlation between elastic and plastic strains in LCF were analyzed and a strain ratio-based fatigue model was proposed and examined using various materials. The main conclusions can be drawn as follows:

(1) Two ways that strain ratio affects the LCF process are found, depending on the strain condition. For low strain cases, the effect of strain ratio on fatigue life relies on the mean stress; for high strain cases, the plastic strain energy controlled by the strain ratio dominates the fatigue life and the mean stress plays a small role. This is why the mean stress-based models struggle in small-amplitude loading cases but show acceptable accuracy in large-amplitude loading conditions;
(2) The corrected fatigue strength coefficient *f*(*R*) and fatigue ductility coefficient *g*(*R*) show linear correlations with the strain ratio *R*, which can effectively represent the complex behavior of the material, including the mean stress relaxation and ratcheting;
(3) Temperature is not considered in the present study, which may play a considerable part in the LCF of metallic and nonmetallic materials. Further work could be done in such directions to further understand the LCF mechanism.

## Figures and Tables

**Table 1:** Comparison of relative errors of different models.

S_z_	Manson–Coffin	SWT	Goodman	Morrow	Walker	Lv	Wang	Proposed
HPTS [19]	1.848	0.706	0.439	0.377	0.362	0.834	0.378	0.247
T851 [16]	0.924	0.163	0.193	0.183	0.184	0.156	0.265	0.152
Resin [22]	198.732	5.225	93.33	14.949	2.519	1.2	0.47	0.467
AZ61A [23]	4.35	1.385	0.775	1.318	0.544	0.239	0.321	0.242

## Nomenclature

*R*	strain ratio
Δ*W*_p_	plastic strain energy
Δ*W*_e_	elastic strain energy
*σ*_m_	mean stress
$\sigma_{f}^{\prime }$	fatigue strength coefficient
*b*	fatigue strength exponent
$\varepsilon_{f}^{\prime }$	fatigue ductility coefficient
*c*	fatigue ductility exponent
*E*	Young’s modulus
*σ*_a_	stress amplitude
*σ*_u_	tensile limit of material
*σ*_eq_	equivalent stress amplitude
*γ*	material constant of Walker model
*α*	material constant of Kwofie model
*β*, *n*	material constant of Wang’s model
$k_{\sigma }$, $k_{\varepsilon }$	material constants of proposed model
*N*_f_	experimental life
*N*_p_	predicted life
*z*	discrepancy of predicted life and experiment life
*S_z_*	discrepancy level of predictions
